# Semi-transparent Perovskite Solar Cells Developed by Considering Human Luminosity Function

**DOI:** 10.1038/s41598-017-11193-1

**Published:** 2017-09-06

**Authors:** Gyu Min Kim, Tetsu Tatsuma

**Affiliations:** Institute of Industrial Science, The University of Tokyo, 4-6-1 Komaba, Meguro-ku, Tokyo, 153-8505 Japan

## Abstract

Semi-transparent solar cells draw a great deal of attention because their applications include, for instance, photovoltaic windows. General approach to semi-transparent cells is using thin active layers or island-type structures. Here we take human luminosity function into account, and develop solar cells that harvest photons in the wavelength regions in which human eyes are less sensitive to light. We used an organic-inorganic hybrid perovskite, which is sensitive to light particularly in the blue and deep-blue regions, and plasmonic silver nanocubes that enhance light harvesting in the red and deep-red ranges. In order to tune the plasmonic wavelength to that range, we took advantage of electrode-coupled plasmons (ECPs). We prepared non-plasmonic semi-transparent solar cells, and reduced the active layer thickness and introduced ECPs, so that the visual transparency index and power conversion efficiency of the cell were improved by 28% and 6%, respectively, of the initial values.

## Introduction

Since the advent of the perovskite solar cell (PVSC), in which organic-inorganic hybrid perovskites are used as an active material, it has attracted great attention because of high power conversion efficiency (PCE) comparable to single junction silicon-based solar cells^1–3^. PVSCs have also been applied to flexible^4–7^ and semi-transparent solar cells^8, 9^ as were other organic solar cells^10–12^. In particular, semi-transparent solar cells can be applied to photovoltaic windows and smart windows.

However, meticulous care is required when developing semi-transparent solar cells using perovskites. In addition to making the top metal electrode semitransparent by using a thin metal layer, metal nanowires^13^, or organic electrode^14^, the transparency of the perovskite layer should also be increased. The mainstream to enhance the transmittance of the perovskite layer is to reduce the amounts of perovskite materials by taking thin active layers^15^ or island-type structures^16, 17^, which inevitably lowers the PCE because of low amounts of active layers.

**Figure 1:**
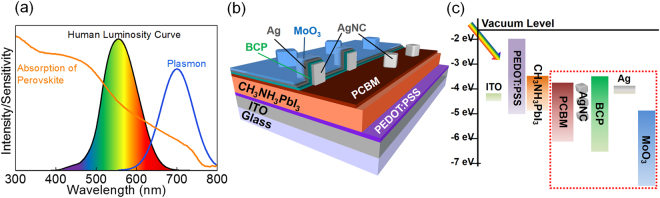
(**a**) Concept of the visual transparency improvement of the semi-transparent solar cells by considering the human luminosity curve. (**b**) The structure and (**c**) the energy diagram of PVSCs with ECPs.

The optical scattering^18^ and the human luminosity factor^19^ should also be taken into account in fabricating semi-transparent PVSCs. Strong scattering from the rough surface of a perovskite layer hinders the visual transparency even if the transmittance measured is high. Nanoscale smoothing of the perovskite surface suppresses the scattering and leads to good visual transparency^18^. The luminosity factor, namely human visual sensitivity to light, is shown in Fig. 1a as a human luminosity curve^19^. It shows that human eyes are sensitive around 550 nm of wavelength, and are less sensitive in the deep blue and deep red ranges. Thus, harvesting photons in those less sensitive regions would improve the efficiency of semi-transparent PVSCs by keeping the high visual transparency.

In order to achieve this, we utilized plasmonic metal nanoparticles (NPs), which have been exploited for enhancement of photocurrents of solar cells including PVSCs^20–22^ by localized surface plasmon resonances (LSPR). There are two principal mechanisms of the plasmonic enhancement, namely the near-field antenna effect, in which light harvesting efficiency is enhanced by optical near field around the NPs^23, 24^, and the far-field scattering effect, in which effective light path length is increased by far-field scattering^25–27^. A less important effect in the enhancement is based on plasmon-induced charge separation (PICS)^28^, in which electron injection from resonant metal NPs to a semiconductor increases the electronic conductivity of the latter^29, 30^. Here we use Ag nanocubes (AgNCs), which exhibit both intense near field at their vertices and strong far-field scattering^31–33^.

In addition, we take advantage of electrode-coupled plasmons (ECPs)^22^, which have been derived from film-coupled plasmons^34–36^. ECPs are the plasmonic coupling between metal NPs and a metal film coated as a top-electrode on the NPs with a thin intervening semiconductor layer between them, as shown in Fig. 1b. It is easy to modulate the ECP wavelength by changing the particle size and the semiconductor layer thickness. Here we developed semi-transparent PVSCs and achieved enhanced photocurrents and PCEs or improved transparency in the wavelength range of high luminosity factor, by adjusting the ECP wavelength to the deep red region, at which the luminosity factor is low (Fig. 1a).

## Methods

### Synthesis of AgNCs

AgNCs were synthesized by a method reported elsewhere with slight modifications^37^. A 5.1 mL of ethylene glycol (EG) in a 20 mL glass vial was stirred with the cap opened for 5 min at 145 °C. NaSH (1.5 mM in EG, 60 μL), HCl (3 mM in EG, 0.5 mL) and poly-(vinylpyrrolidone) (PVP, 20 mg mL^–1^ in EG, 1.25 mL) were injected into the solution successively. CF_3_COOAg (62.3 mg mL^–1^ in EG, 0.4 mL) was injected after a 2 min interval. The solution was stirred (180 min) until the size of AgNCs reaches 70 nm. Then suspension was washed with acetone and water twice. After centrifugations, the 70 nm of AgNCs were dispersed in methanol (5 mL).

### Preparation of semi-transparent PVSCs

A patterned indium–tin oxide (ITO) electrode was pre-treated in deionized water, acetone and 2-propanol, followed by an oxygen plasma treatment (40 s) to obtain a hydrophilic surface. Poly(3,4-ethylenedioxythiophene) and poly(styrenesulfonate) (PEDOT:PSS, Clevios AI 4083, 50 μL) was spin-coated at 5000 rpm for 30 s, followed by annealing at 150 °C for 10 min. The perovskite layer was processed by a short-spinning and vacuum drying (SSVD) method, which we previously reported for ultrasmooth perovskite films^18^. The perovskite precursor of 3:1 ratio of CH_3_NH_3_I and PbCl_2_ in dimethylformamide (28 wt% for 180 nm-thick and 32 wt% for 210 nm-thick perovskite layer) was spin-coated at 4000 rpm for 5 s on PEDOT:PSS under 50% of relative humidity. Then, the substrate was promptly transferred to a vacuum oven, in which the wetted film was dried at 0.05 kPa for 15 min, followed by annealing at 60 °C for 25 min and 65 °C for 5 min. The substrate was taken out to the ambient air and coated with phenyl-C_61_-butyric acid methyl ester (PCBM, 1.8 wt % in chlorobenzene, 30 μL) at 3000 rpm for 45 s. A 15-μL aliquot of the AgNC suspension was dynamically dispensed on the PCBM layer while the substrate was spinning at 4000 rpm to reach 2% coverage of AgNCs on PCBM. 10 nm of bathocuproine (BCP), 10 nm of Ag and 10 nm of MoO_3_ were deposited sequentially by evaporation onto AgNCs as transparent electrodes.

### Characterization

Optical properties of the samples were measured by using a spectrophotometer (Jasco V-670) equipped with an integrating sphere. We obtained photocurrent action spectra using Hamamatsu Photonics OSG as a light source (photon flux = 5 × 10^15^ photons cm^−2^ s^−1^). The current density–voltage characteristics were measured by scanning the voltage at 100 mV s^−1^ with a source meter (Keithley 2612B) under AM1.5 G irradiation (100 mW cm^–2^) from a solar simulator (Bunkoukeiki, BSS-150T).

### Spectral simulation

Optical spectra and electric field distributions were simulated by a finite-difference time-domain (FDTD) method using FDTD Solutions (Lumerical Solutions). The simulation domain (400 × 400 × 400 μm) consisted of 4 nm cubic cells, and the central region (130 × 130 × 130 nm) around a AgNC was further meshed with a three-dimensional grid of 1 nm spacing. Backward scattering from the nanocube was monitored by a 340 × 340 nm square screen set 120 nm apart from the AgNC–PCBM interface. The dielectric functions of Ag, glass and MoO_3_ were extracted from literature data^38^. Refractive index values of PCBM and BCP were 2 and 1.7 respectively.

## Results and Discussion

### Far-Field Scattering Effects

Figure 1b,c shows the structure and the energy diagram of the semi-transparent PVSCs that we developed. The BCP/Ag/MoO_3_ multilayer (10 nm thick each) was used as a transparent electrode for the present planar type PVSCs by taking advantage of high transparency with low reflection, which occurs when materials with high refractive index (*n*) (BCP and MoO_3_ in this case) are coated on both sides of a material with low *n* such as Ag under certain conditions^39–41^. The present transparent electrode can also be coupled electromagnetically with AgNCs in contact with BCP to generate ECPs.

**Figure 2:**
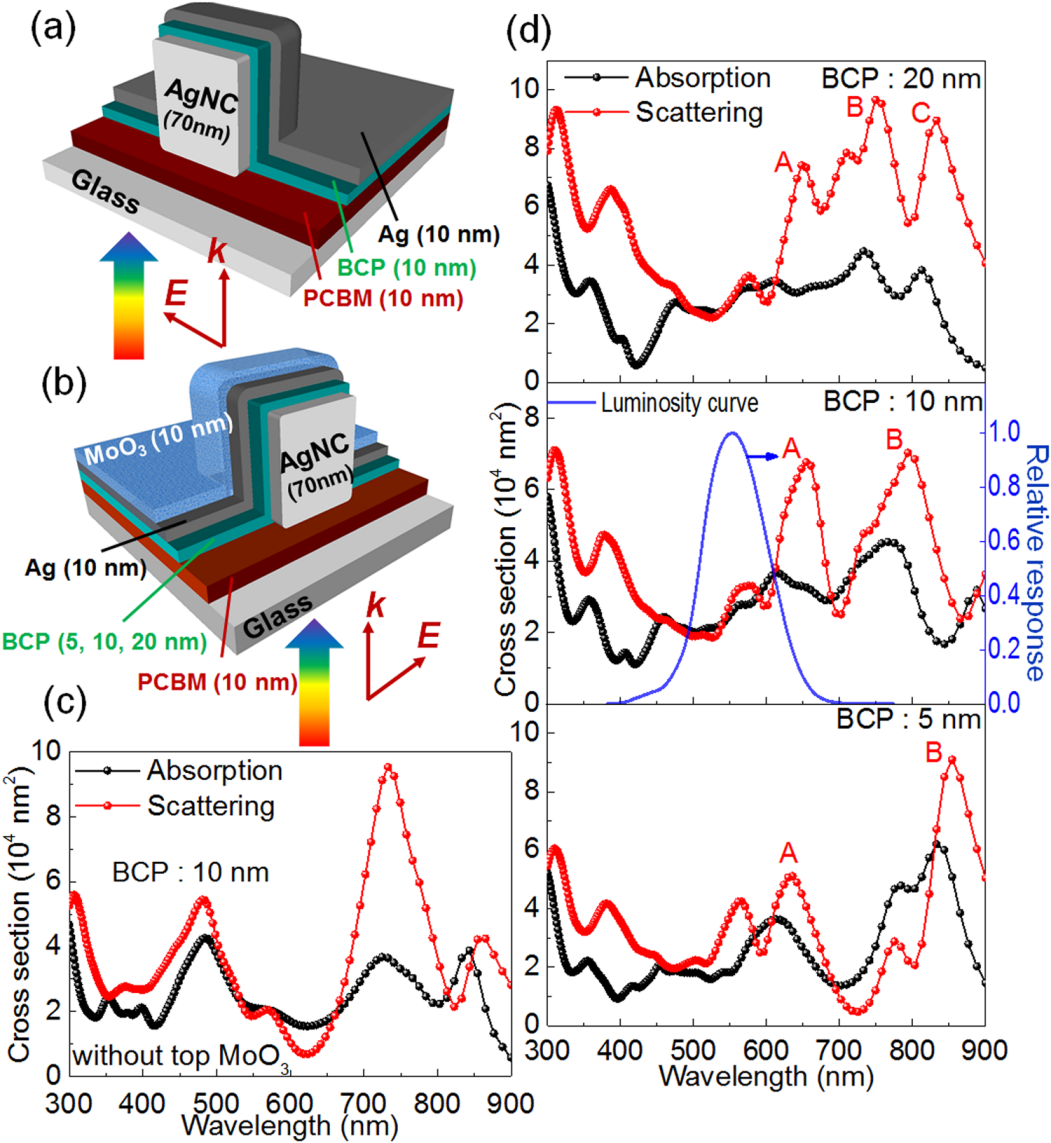
Models for the FDTD simulation (**a**) without and (**b**) with the top MoO_3_ layer. (**c**) Simulated scattering and absorption spectra for the model shown in (**a**). (**d**) Those for the model in (**b**). The human luminosity curve is also drawn in (**d**).

In order to confirm the effectiveness of ECPs in the transparent PVSCs, FDTD simulation was conducted. In our previous work regarding the ECP for AgNCs coupled with a thick, non-transparent Ag electrode (100 nm thick) through BCP, the optimum BCP thickness was 10 nm, with which the ECP appeared at ~760 nm^22^. This resonance is red-shifted from that of a AgNC without electrode-coupling (525 nm)^22^ by 235 nm. Here, we examined coupling of AgNCs with a thin, transparent Ag electrode of 10 nm thick, using the model glass/PCBM/AgNC/BCP/Ag shown in Fig. 2a. The PCBM layer is also 10 nm thick. Here we assume that the refractive index of the perovskite layer is close to that of glass. In the simulated spectra, the ECP appears at ~735 nm (Fig. 2c). The red-shift from the resonance without electrode-coupling (by 210 mn) is smaller than that for the coupling with the thick electrode (by 235 nm). This can be explained in terms of slightly weaker coupling of AgNCs with the thinner Ag electrode.

Then we coated the thin Ag electrode with a 10-nm-thick MoO_3_ layer (Fig. 2b) to improve the transparency as described above. The calculation based on this model gives two large peaks (Peaks A and B) at ~650 and ~790 nm (Fig. 2d). This is likely due to high refractive index of MoO_3_, which is ~2.5, as the LSPR peaks generally red-shift as the local refractive index increases. We also calculated for the models with different BCP thicknesses, 5 and 20 nm (Fig. 2d). When the BCP layer is 20 nm thick, one more peak (Peak C) appears at ~830 nm. As the BCP layer thickness is decreased, Peaks B and C red-shift while Peak A stays at ~650 nm. Thus, Peak A is not based strongly on ECP, whereas Peaks B and C are ascribed to ECPs. In Fig. 2d, the human luminosity curve is also shown. It is obvious that the overlapping of the plasmonic absorption and scattering with the luminosity curve is mitigated as the BCP thickness increases. In addition, the ratio of the scattering intensity to the absorption intensity increases with the BCP thickness. Therefore, it is expected that the far-field scattering effect enhances and thermal energy dissipation via the plasmonic absorption by AgNCs decreases as the BCP is thickened.

From the optical point of view, 20-nm-thick BCP is the most appropriate among the BCP layers examined, for the plasmonic enhancement in the wavelength range where human eyes are not very sensitive. However, the serial resistance of the cell with 20-nm-thick BCP layer was higher than that of the cell with 10-nm-thick BCP by a factor of 2.7, suggesting that a Schottky barrier might form at the interface between Ag and the 20-nm-thick BCP layer. We therefore selected the 10-nm-thick BCP layer for construction of semi-transparent PVSCs.

### Near-Field Antenna Effects

**Figure 3:**
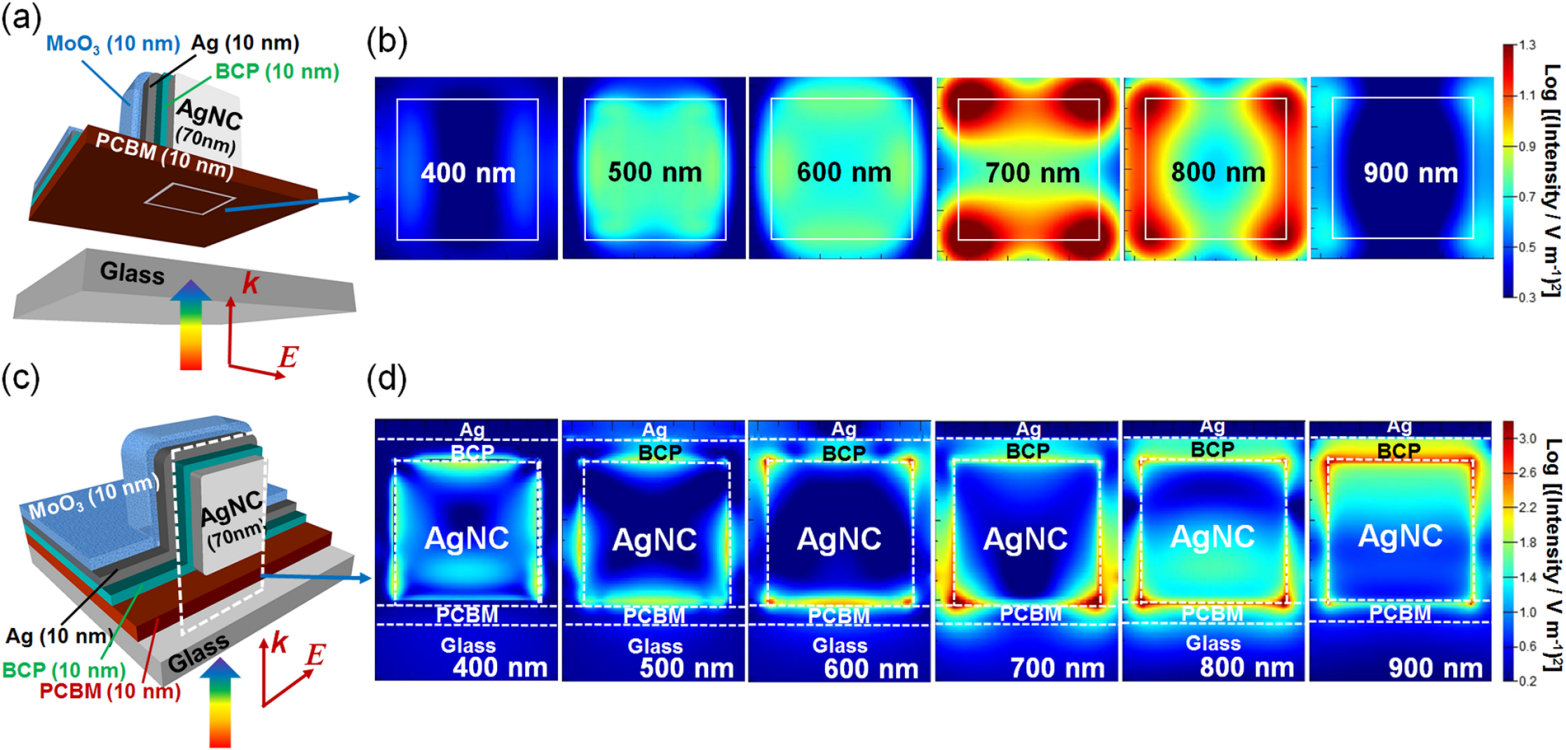
Schematic illustrations of the (**a**) lateral and (**c**) vertical planes at which the electric field distributions are monitored. (**b**) Lateral (at the PCBM-glass interface) and vertical (**d**) distributions of the plasmonic electric field calculated by the FDTD method.

We also calculated the electric field distributions at the PCBM-glass interface on the basis of the same model used for the transparent electrode with 10-nm-thick BCP (Fig. 3a), so as to see the near-field antenna effects of the ECPs on the perovskite layer. Note that the glass is replaced with a perovskite layer in the experimentally prepared real PVSCs. The results calculated at several different wavelengths are shown in Fig. 3b. The electric field penetrates through the 10-nm-thick PCBM layer in the wavelength range 500–900 nm, and is strong in particular at 700–800 nm range. From the cross-sectional distributions in the dashed white square region depicted in Fig. 3c, it is obvious that the electric field is strongly localized at the bottom face of the AgNC, which is the face close to the perovskite in the real cell, in the 700–800 nm range (Fig. 3d).

### Optical Properties of the Experimentally Prepared Cells

**Figure 4:**
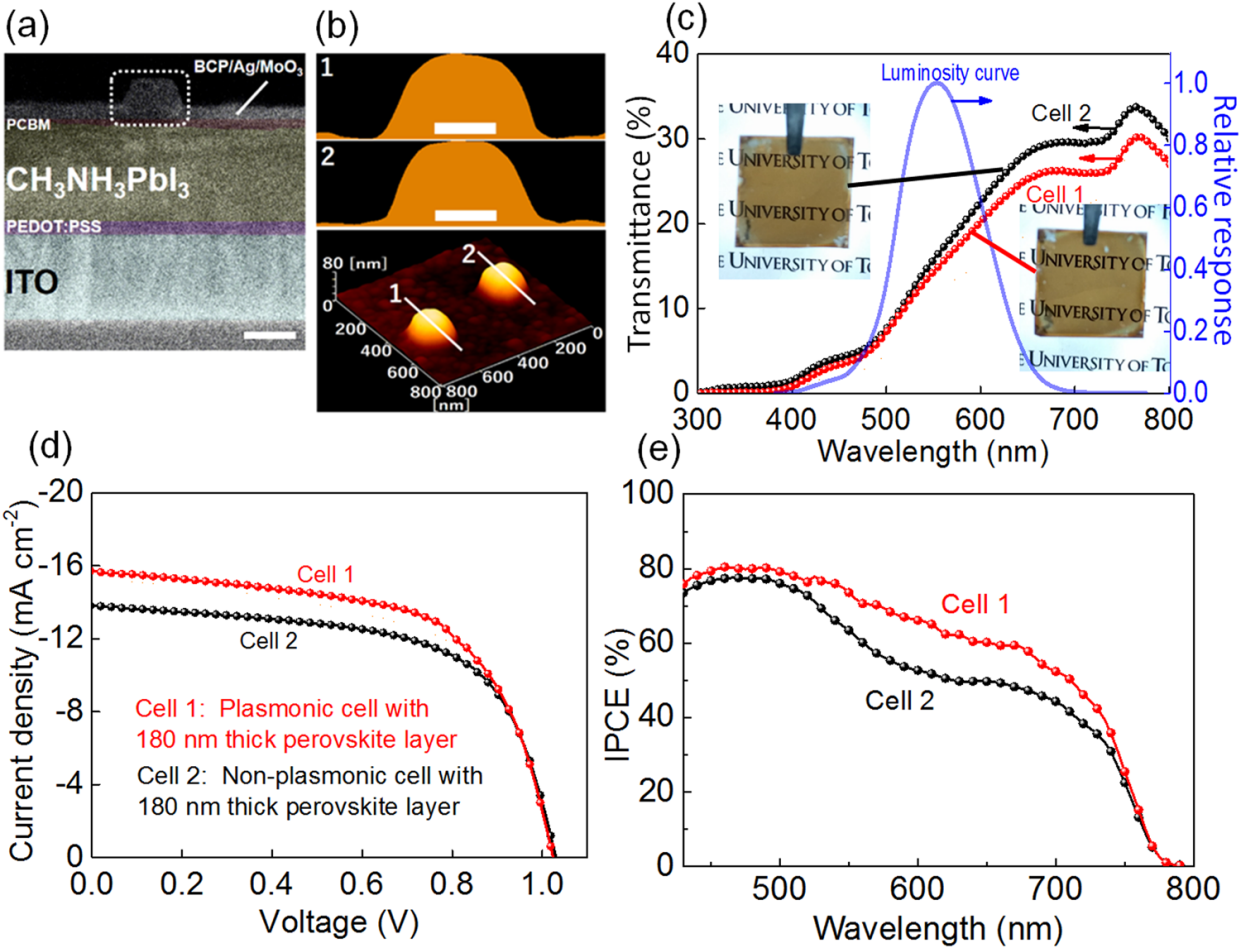
(**a**) SEM (scale bar is 100 nm) and, (**b**) AFM images of the plasmonic semi-transparent PVSCs; the height profiles of the latter (scale bar is 50 nm) are for lines 1 and 2 of the three-dimensional image (bottom). (**c**) Transmittance spectra, (**d**) *J*-*V* characteristics and (**e**) IPCE action spectra of the plasmonic and non-plasmonic PVSCs (Cell 1 and Cell 2, respectively), together with the human luminosity curve and photographs of the cells in (**c**). The perovskite layers are ~180 nm thick.

We fabricated the semi-transparent PVSCs with the structure shown in Fig. 1b. For a better visual transparency, a thin perovskite layer (~180 nm) is used instead of a typical thickness (~300 nm) in non-transparent PVSCs. In our previous work on ECPs, the optimum coverage of AgNCs was 2% because higher coverage of AgNCs are detrimental not only to the cell transparency but also to the cell performances because they work as electron trap sites^22^. Although the latter effect could be suppressed by coating the AgNCs with a more insulating layer like SiO_2_, it would elongate the distance to the Ag electrode and that to the perovskite layer, resulting in lower ECP intensities and weaker near-field effects, respectively. Figure 4a shows a cross-sectional SEM image of the semi-transparent PVSCs. The electrode keeps the shape of AgNCs (shown in the dashed white square) even after covering with BCP, Ag and MoO_3_ (10 nm thick each), as confirmed also by atomic force microscopy (Fig. 4b).

The transmittance spectra of the plasmonic semi-transparent PVSC with AgNCs (Cell 1) and non-plasmonic semi-transparent PVSC without AgNCs (Cell 2) are shown in Fig. 4c. From those spectra, we evaluated the optical properties of the cells in terms of the average transmittance (AVT) (eq. 1), which is often used as an index for transparency, and the visual transparency index (VTI), which we define by eq. 2 as an improved index for the visual transparency:

1 $$\mathrm{AVT}\,( \% )=\,\frac{{\int }_{400}^{800}T(\lambda )d\lambda }{(800\,-\,400)}$$

2 $$\mathrm{VTI}\,( \% )=\,\frac{{\int }_{400}^{800}[T(\lambda )\,RLF(\lambda )]d\lambda }{{\int }_{400}^{800}RLF(\lambda )d\lambda }\,$$

where *λ* is the wavelength, *T*(*λ*) (%) the transmittance at *λ* and *RLF*(*λ*) the relative luminosity factor at *λ* plotted in the luminosity curve, as listed in Table 1. The AVT value of the plasmonic PVSCs (Cell 1, 17.8%) is smaller than that of the non-plasmonic ones (Cell 2, 19.8%) by 10% of the latter value because of the absorption and scattering of ECPs in the latter cell. However, since AVT is the simple average of the transparency in the 400–800 nm range, it is not appropriate for evaluation of visual transparency. We therefore evaluated VTI values (Table 1), in which the transparencies at different wavelengths are weighted by considering human luminosity function as eq. 2 indicates. The VTI value of the non-plasmonic PVSCs is decreased by the introduction of AgNCs only by 4%. This is because the ECP resonance occurs mostly at >600 nm, almost out of the luminosity curve peak (500–600 nm). Actually, the visual transparency of the plasmonic PVSCs (Cell 1) is only slightly lower than that of the non-plasmonic cells (Cell 2) as confirmed by the photographs (insets of Fig. 4c).

**Table 1:** Performances of the semi-transparent PVSCs with and without ECPs under AM 1.5 G light irradiation.

Cell	ECP	Thickness (nm)	*J*_sc_ (mA cm^−2^)	*V*_oc_ (V)	FF	PCE (%)	AVT (%)	VTI (%)
1	ECP	~180	15.69 ± 0.11	1.02 ± 0.0046	0.60 ± 0.0074	9.73 ± 0.12	17.8	15.6
2	─	~180	13.48 ± 0.38	1.02 ± 0.0079	0.61 ± 0.012	8.62 ± 0.24	19.8	16.3
3	─	~210	15.34 ± 0.35	1.00 ± 0.0043	0.60 ± 0.0098	9.18 ± 0.19	15.2	12.2

All the values (mean ± standard deviation) were evaluated for at least four cells. The light intensity was 100 mW cm^−2^.

### Photovoltaic Performances of the Cells

The current density-voltage (*J-V*) characteristics in Fig. 4d show that the plasmonic semi-transparent PVSCs with ~180-nm-thick perovskite layer (Cell 1) exhibit higher PCE value of 9.73 ± 0.12% compared to the non-plasmonic cells (Cell 2, 8.62 ± 0.24%). The PCE value is increased by 13% of the original value by introduction of AgNCs, whereas the VTI value is decreased only by 4%. These results clearly indicate the effectiveness of our concept, in which human luminosity curve is considered.

The increase in PCE by introducing AgNCs stems from the enhanced short-circuit current (*J*_sc_) (from 13.48 ± 0.38 mA cm^−2^ to 15.69 ± 0.11 mA cm^−2^) while open-circuit voltage (*V*_oc_) and fill factor (FF) show no significant changes. Incident photon-to-current conversion efficiency (IPCE) reveals that the enhancement of *J*_sc_ is caused by the photocurrent increase in the wavelength range 520–750 nm. This comes from the far-field scattering effect and the near-field antenna effect of ECPs whereas the range is somewhat blue-shifted from the wavelength range in which strong scattering ( ≥600 nm, Fig. 2d) and near field ( ≥700, Fig. 3) are expected and enhanced extinction is observed ( ≥600 nm, Fig. 4c). A certain portion of the enhancement could therefore be explained in terms of improved electronic conductivity in the BCP layer as a result of electron injection from the AgNCs due to PICS^28–30^. It is expected that PICS occurs more efficiently at shorter wavelengths, because energy of photons are higher and more advantageous to get across the energy gap at the BCP-AgNC interface. Actually, the serial resistance is decreased by ~7% by introduction of AgNCs as evaluated from the *J-V* curves. Since the shunt resistance is also decreased as shown in Fig. 4d, the FF value is slightly decreased.

**Figure 5:**
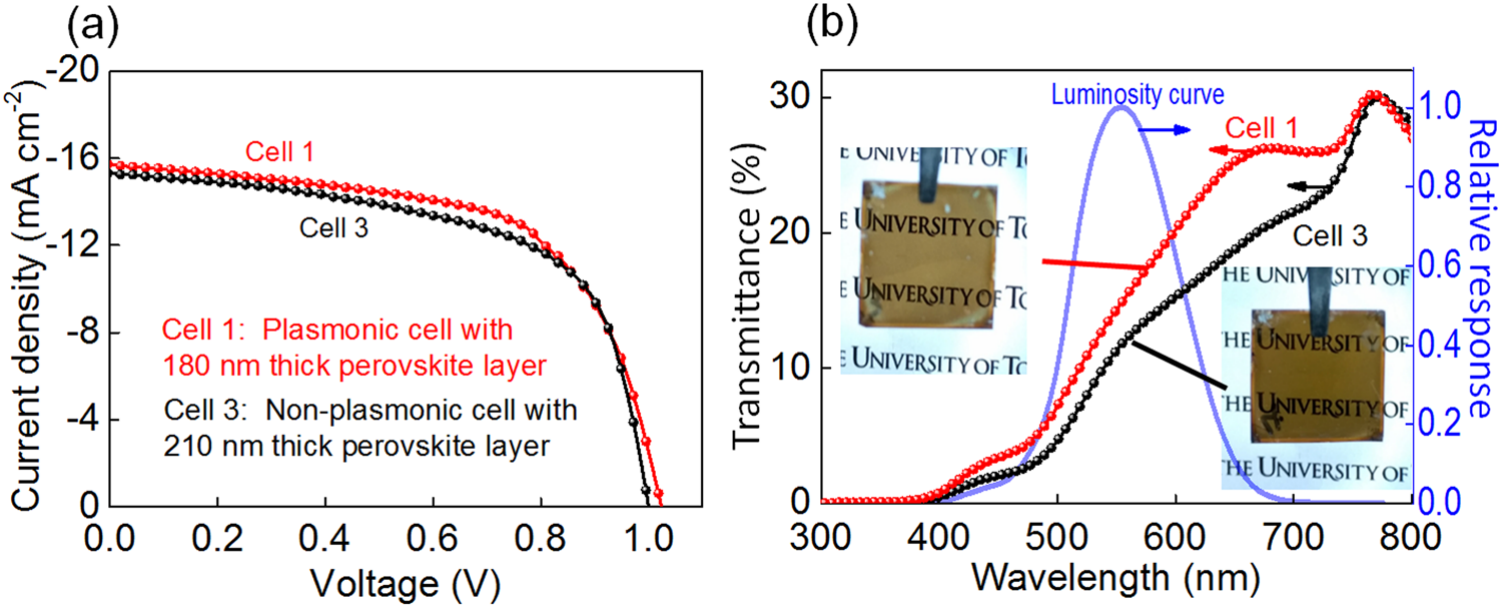
(**a**) *J*-*V* characteristics, (**b**) transmittance spectra and the photographs (insets) of the plasmonic PVSC with ~180-nm-thick perovskite layer and the non-plasmonic PVSC with ~210-nm-thick perovskite layer together with the human luminosity curve.

Since the photocurrent increment upon introduction of AgNCs is 16.4%, we thickened the perovskite layer of the non-plasmonic cell from 180 ± 20 nm to 210 ± 20 nm so as to roughly match the photocurrents to those of the plasmonic cell with a ~180-nm-thick perovskite layer. As a result, the non-plasmonic PVSCs with the thicker perovskite layer (Cell 3) gave the *J*_sc_ value of 15.34 ± 0.35 (mA cm^−2^), which is in close agreement with that of the plasmonic cell with the thinner perovskite layer (Cell 1), and slightly decreased *V*_oc_ (Fig. 5a, Table 1). This is in accordance with our previous data^18^ that the *V*_oc_ value decreases with increasing perovskite layer thickness. The transmittance of Cell 3 is lower than that of Cell 1 in the whole wavelength range examined (Fig. 5b), and the AVT and VTI values of Cell 1 are higher than those of Cell 3 by 17% and 28%, respectively, of the values for Cell 3 (Table 1). Accordingly, the visual transparency shown in the photograph is also lower for the non-plasmonic cell than that for the plasmonic cell (insets of Fig. 5b). These results indicate that ECPs are effective for improvement of the photovoltaic performances with keeping the visual transparency, or for increasing the visual transparency with retaining the photovoltaic performances.

Since the cells were not encapsulated, the performances were degraded gradually by exposure to air and moisture, likely because of deterioration of the perovskite layer and AgI formation of the Ag top electrode^42^. However, no significant difference in the stability was observed between the plasmonic and non-plasmonic cells. Adequate encapsulation of the cells would improve the stability^43^.

## Conclusions

We developed high performance semi-transparent PVSCs by taking advantage of the plasmon coupling between AgNCs and the thin transparent electrode (ECPs) taking human luminosity function into account. When ECPs were simply introduced to a semi-transparent PVSCs, the PCE was improved by 13% while VTI was decreased by 4% of the initial values. If the perovskite layer thickness was decreased and ECPs were introduced, the PCE and VTI values were improved by 6% and 28%, respectively, of the initial values. Therefore, ECPs are promising in improvement of the performances of semi-transparent solar cells.
